# Electroanalytical Sensors and Devices for Multiplexed Detection of Foodborne Pathogen Microorganisms

**DOI:** 10.3390/s90705503

**Published:** 2009-07-13

**Authors:** María Pedrero, Susana Campuzano, José M. Pingarrón

**Affiliations:** Departamento de Química Analítica. Facultad de Ciencias Químicas. Universidad Complutense de Madrid. E-28040 Madrid, Spain; E-Mails: mpedrero@quim.ucm.es (M.P.); susanacr@quim.ucm.es (S.C.)

**Keywords:** pathogen microorganisms, multiple detection, electrochemical sensors

## Abstract

The detection and identification of pathogen microorganisms still rely on conventional culturing techniques, which are not suitable for on-site monitoring. Therefore, a great research challenge in this field is focused on the need to develop rapid, reliable, specific, and sensitive methods to detect these bacteria at low cost. Moreover, the growing interest in biochip development for large scale screening analysis implies improved miniaturization, reduction of analysis time and cost, and multi-analyte detection, which has nowadays become a crucial challenge. This paper reviews multiplexed foodborne pathogen microorganisms detection methods based on electrochemical sensors incorporating microarrays and other platforms. These devices usually involve antibody-antigen and DNA hybridization specific interactions, although other approaches such as the monitoring of oxygen consumption are also considered.

## 1. Introduction

The rapid and reliable detection and determination of pathogen microorganisms is of great importance nowadays, due to health and safety reasons. The main areas of research implied in this subject are the food industry, water and environment quality control and clinical diagnosis [1]. Among these, the food industry is the area where most attention has been focused, given the public health implications and potential fatal consequences of failing to detect certain bacteria while it is still possible to take direct action. Pathogens belonging to the coliforms, salmonellae, bacilli, etc. families that have been used in bioterrorism attacks aimed at the food supply [2] should be considered. As an indication of the subject relevance, it can be mentioned that six reviews have been published only in the 2008–2009 period, dealing with electrochemical biosensors for food pathogens [3], applications of microarrays in pathogen detection and biodefence [4], traditional pathogen detection methods and biosensors [5], on-site pathogen detection using antibody-based sensors [6], and electrochemical impedance for rapid detection of foodborne pathogenic bacteria [7].

An analysis of foodborne outbreak data (events in which two or more people became ill after consuming a common food or meal) reported internationally has recently been described by Greig and Ravel [8]. Using outbreak data for food attribution is the only methodological approach where there is an actual direct link between the pathogen, its source and each infected person. As a conclusion of this work, some specific associations were found for foodborne outbreaks that occurred between 1988 and 2007: *Salmonella enteriditis* outbreaks occurred relatively often in the EU states with eggs as the most common source; *Campylobacter* associated outbreaks were mainly related to poultry products in the EU and to dairy products in the US; there was an association between *Escherichia coli* outbreaks and beef in Canada; and while *Salmonella typhimurium* outbreaks were relatively common in Australia and New Zealand, across all regions, *Salmonella* was associated with a variety of food groups. It is clear that diseases caused by foodborne pathogens have been a serious threat to public health and food safety for decades and remain one of the major concerns of our society. It has captured the attention, not only of the scientific community, food industry or the academia, but also of the public, that has become increasingly aware and concerned about the health risks posed by the foodborne pathogens [7]. The major economic and social importance of food quality and safety in the EU policy is due to the fact that foodborne illness occurring each year in Europe costs hundreds of millions of Euros, while in the USA it has been estimated that more than 36 million cases of illness occur annually because of foodborne and waterborne pathogens [5].

As a consequence, there is a widely felt need to develop methods for the early identification of emerging hazard to food safety with the aim of preventing these hazards from becoming real risks and causing incidences. Kleter *et al*. [9] have reviewed various international projects dedicated to the early identification of hazards (SAFE FOODS sponsored by the European Commission Directorate for Research’s Sixth Framework Program, EMRISK funded by the European Food Safety Authority, etc.). Trends in data generated by surveillance may provide indicators of the emergence of certain pathogens based on trends towards increased incidences. An example of such a surveillance program is PulseNet, a collaboration of US state public health laboratories which also cooperates with several laboratory networks in Europe, Canada, Japan and other Asian and Latin American countries in the research on outbreaks of several pathogen microorganisms. Time-analysis saving methods able to rapidly identify the pathogen microorganism causing an outbreak is of great importance for this kind of monitoring and electrochemical multiplexed sensor systems, which would also be sensitive, accurate, simpler and cheaper than other existing systems would be of great help in this kind of tasks.

Correct detection and identification of foodborne pathogens based on conventional culturing techniques are very laboring, time-consuming, and have to be completed in a microbiology laboratory, so that it is not suitable for food quality assurance to make timely response to possible risks. Thus, miniaturized biochemical tests, physicochemical methods that measure bacterial metabolites, highly specific nucleic acid-based tests, antibody-based methods and fully automatic instrumental diagnostic systems have been used for this purpose [7]. In fact, various biosensors for pathogenic bacteria detection with improved analysis time, sensitivity and reliability have been described [10–14].

Biosensor-based tools offer the most promising solutions, electrochemical biosensors having the advantage of high sensitivity, rapidity, low cost and amenability towards micro-fabrication [3]. An ideal biosensing platform should meet the requirements of miniaturization, cost-efficiency and ability for simultaneous detection of multiple analytes. The constant demands for more sensitive, accurate, and faster analytical procedures have, in fact, led to miniaturized and multiplexed assays. One of the advantages of micro- and nano-fabrication techniques in the field of biosensors is the possibility of achieving one shot multi-analyte analysis with the subsequent shortening of the analysis time [1]. Although automation in food pathogen detection methods is highly desirable, to date an ideal rapid and automated system joining a high throughput format, differentiation of live and dead cells, low cost, simplicity and accuracy, does not exist.

As a consequence of the large number and diversity of microbial pathogens and their virulence factors, an increase in the interest on technologies capable of detecting multiple pathogens and virulence factors simultaneously has been observed lately. Moreover, nowadays, an effective microbial detection system should be able to simultaneously detect multiple pathogen and toxins, and to distinguish them from related species and virulence factors. In this sense, this review will be focused on the development of electrochemical multiplexed pathogen sensors, mainly DNA and immunosensors.

## 2. Electrochemical Immunosensors

Electrochemical immunosensors developed for simultaneous multiplexed analysis of pathogenic bacteria use mostly electrochemical impedance spectroscopy as the transduction technique, thus providing label-free, on-line and high throughout devices for bacteria detection. Impedance biosensors for bacteria detection are based on the measurement of changes in the electrical properties of bacterial cells when they are attached to or associated with the electrodes [3,7]. Moreover, the advances in microfabrication techniques have enabled the use of microfabricated microarray electrodes for impedance detection and the miniaturization of impedance microbiology into a chip format.

As early as 1998, Louie *et al*. [15] described the development of an impedance-based field biosensor system for the detection of the foodborne pathogens *E. coli* O157:H7 and *Salmonella* spp. The portable biosensor system used a variety of specific sensor modules, each of which could be used to quantitatively measure the presence of specific analytes. The complete device comprised: (1) a proprietary immobilization and stabilization technology that retained bioactivity and provided stability for extended storage, (2) an interdigitated differential binding module design using gold electrodes on a silica chip allowing for simultaneous direct measurement of sample and reference binding events, and (3) an electronics module to quantitatively measure analyte binding to the disposable module. Different approaches were assayed for the biosensor module operation, including an antibody-based system with anti-*E. coli* O157:H7. The response for each sensor was rapid, and stable readings could be obtained in less than 1 min. However, although a portable, reagentless immunosensor technology was described allowing for rapid detection of specific pathogens, no real sample application was considered.

**Figure 1.**
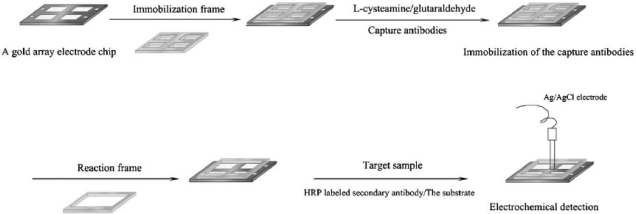
Schematic illustration of impedance multiplexed detection of rat IgG, HBsAg and HBeAg (Yu *et al*. [16]).

A method based on electrochemical impedance spectroscopy (EIS) combined with a gold electrode array was developed by Yu *et al*. [16] to assay a mixture of rat IgG, Hepatitis B virus surface antigen (HBsAg) and Hepatitis B e antigen (HBeAg). As shown in Figure 1, the gold array electrode chip consisted of four Au working electrodes, and self-assembled monolayers (SAMs) of 2-mercaptoethylamine were formed on each electrode, followed by the addition of glutaraldehyde. Then, the resulting electrode surface with aldehyde groups readily coupled with amino groups of the capture antibodies. BSA was used to deactivate the excess aldehyde groups and block the electrostatically bound proteins. Then, the target sample was added followed by HRP-labeled secondary antibodies, and the enzymatic precipitation response to the addition of 3-amino-9-ethyl carbazole (AEC) as substrate was measured. This impedance detection approach based on the enzyme-label method not only increased detection sensitivity when compared with ELISA kits, allowing a limit of detection of 10 pg mL^−1^ for HBsAg, but also prevented a crossover phenomenon on the array.

In spite of the possibilities offered by electrochemical immunosensors in terms of specificity, sensitivity and also rapidity when compared with classical immunoassays, and of the significant progress that has been made in the development of detection platforms based on recent advances in microfabrication and electromechanical technologies, to date not much work has been described on the application of this kind of sensors to the development of multiplexed analysis methods for pathogenic bacteria. Still, it is foreseeable that the successful integration of micro- and nano-fluidics with immunosensors will result in the development of low-cost and easy fabrication immunosensor electrochemical assays for multiplex analysis of different pathogen microorganisms in real samples.

## 3. Electrochemical DNA Sensors

Electrochemical DNA sensors are based on the immobilization of a single-chain DNA strand onto an electrode and the measurement of changes in electrical parameters caused by the hybridization reaction. One of the major trends in the research of novel diagnostic systems is the development of DNA screen-printed microarrays to produce high dense microband sensor arrays coated with different probes for simultaneous detection of multiple DNA-target sequences [17].

The ability to selectively immobilize different oligonucleotide probes coupled with a sensitive electrochemistry-based detection for multiple species utilizing the preferential catalytic silver electrodeposition process on DNA-linked nanogold shells was reported in 2004 by Cai *et al*. as an important step forward for the realization of a portable bioanalytical microdevice for the rapid detection of pathogens [18]. The methodology used an indium tin oxide (ITO)-coated glass patterned to produce four circular working electrodes. A potential-dependent electropolymerization step was used to selectively address each individual electrode with specific DNA probes. Firstly, two diagonal spots were modified by the polymer scopoletin embedded with streptavidin. Then, a solution of biotin-tagged *Stachybotrys chartarum* (an airborne pathogen) DNA probe was pippetted to the four ITO electrodes. The streptavidin embedded within the electro-deposited polymer matrix allowed a selective immobilization of the DNA probes on the electrodes to which the voltage was applied. After washing unbound DNA molecules, a similar procedure was applied to immobilize *Escherichia coli* probes on the other two remaining spots of the ITO electrode. Recognition of the DNA hybridization events between the immobilized probes and the target pathogen PCR products was achieved through the binding of gold nanoparticle labels to the hybridized PCR amplicons followed by the deposition of metallic silver. The amount of silver deposited onto the gold nanoparticle label was determined by potentiometric stripping analysis (PSA) measuring the oxidative silver dissolution response. The developed methodology applicability was only tested in the detection of PCR amplicons, and therefore this work can be considered as a first step towards the design of a portable DNA analyzer where sample preparation and micro-PCR functionalities should still be developed and integrated.

**Figure 2.**
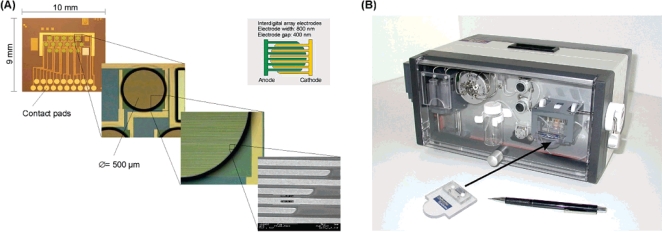
(A) Electrical biochip microarray design and detailed zoom view. (B) Fully automated eBioChip Array analyzer “eMicroLISA” with fluidic, rotor valve, reagent reservoirs, and one hand plug and play ChipStick. Reproduced from Elshoz *et al*. [13].

Low-density electrical 16S rRNA specific oligonucleotide microarrays coupled to an automated analysis system (Figure 2) were developed by Elshoz *et al*. [13] for the identification and quantization of five pathogens typically involved in urinary tract infections. Interdigitated gold array electrodes (IDA-electrodes), with dimensions in the nanometer range, were used for sensitive analysis (limit of detection for *Escherichia coli* total RNA, 0.5 ng μL^−1^). Five different capture probes (thiol-modified oligonucleotides) were spotted on the electrodes, each of them onto three of the array positions. Additionally, three unlabeled oligonucleotides were hybridized in close proximity to the capturing site. These acted as supporting molecules because they improved the RNA hybridization at the capturing site. A biotin labeled, at the 3′ end, detector oligonucleotide was also hybridized to the captured RNA sequences. The biotin labels enabled the binding of avidin alkaline phosphatase conjugates. The enzyme liberated the electrochemical mediator *p*-aminophenol from its electrically inactive phosphate derivative.

The electrical signals were generated by amperometric redox cycling, applying a potential of +350 mV to the anodic fingers and −150 mV to the cathodic fingers of the IDA electrodes, and detected by a unique potentiostat. The readout signals of the microarray were position specific and changed over time proportionally to the analytes concentration. The control of fluidics for variable assay formats, as well as the multichannel electrical readout and data handling were all fully automated. The fast (25 min) and easy procedure did not require any amplification of the targeted nucleic acids by PCR. Five different pathogens, *Escherichia coli*, *Pseudomonas aeruginosa*, *Enterococcus faecalis*, *Staphylococcus aureus*, and *Staphylococcus epidermidis*, were quantified, the discrimination of species from a mixture of pathogens having been also shown.

Further on, in order to develop this methodology and explore its potential, different parameters influencing this chip-array based electrical detection of DNA for analysis of pathogenic bacteria were analyzed using enteropathogenic *Bacillus cereus* as a model, and its toxin-encoding genes as targets [19]. The studied parameters were: (1) rehydration of capture probe layer of functionalized chip arrays and efficient hybridization of targets irrespective of their length, which resulted in signal enhancement when high-ionic-phosphate-buffered saline was used; (2) placement of two adjacent capture and detection probe-binding sites at a terminal part of the target strand, which resulted in significant signal increase; (3) ultrasonic fragmentation of targets for up to 10 min amplified the signals up to two fold for longer DNA strands (> 300 bp), no obvious effect being observed for shorter than 400-bp PCR amplicons, while more than 10 min ultrasonication diminished the specific electrical responses for DNA strands of all sizes; (4) no benefits in assay sensitivity were recognized by the use of longer capture probe linkers than a 6-C linker; and (5) target analytes were detected with discrimination against mismatches even for single nucleotide sequence alteration.

The electrical microarray biosensing system was used for the recognition of PCR amplified gene segments of four pathogens which are among the biowarfare agents of the highest threat potential: *Bacillus anthracis* (BA), *Yersinia pestis* (YP), *Francisella tularensis* (FT) and ortho pox viruses (OPV) [20]. The biointerface was built up with thiol-modified capture oligonucleotides on gold, and functioned as an advanced screening method for the parallel detection of a panel of the four pathogens. The fully automated analysis could be carried out in 27 min, the device having been claimed to be useful for on-site or point-of-care detection in molecular diagnosis. Although this is a robust and simple to use technique, its validation and application to the determination of these microorganisms in real samples has not been reported yet.

Gold and graphite disposable screen-printed electrode arrays were used by Laschi *et al*. for simultaneous electrochemical measurements of the hybridization reaction [21]. Some of the disadvantages encountered with serial arrangements of the working electrodes, such as the maintenance of a potentiostatic control over electrodes due to ohmic drop, or the occurrence of chemical cross-talk when the product from an upstream electrode causes non-specific responses on a downstream electrode, can be reduced by the radial positioning of electrodes in an array. Thus, the authors used a radial configuration array where a silver pseudo-reference electrode was surrounded by four working electrodes. Oligonucleotide sequences, codifying for the *Listeria monocytogenes* toxin inlA were used. Each electrode in the array was modified using thiol-tethered oligonucleotide probes. The target sequences were captured at the sensor interface via sandwich hybridization with surface tethered probes and biotinylated signaling probes. The resulting biotinylated hybrids were coupled with a streptavidin-alkaline phosphate conjugate and then exposed to a α-naphthyl phosphate solution. Differential pulse voltammetry (DPV) was used to detect the α-naphthol electrooxidation signal. The analytical performance of the gold electrode array genosensor was better than that using graphite electrodes due to a higher hybridization efficiency. The genosensor was successful for simultaneous analysis of four different samples, or four different analytes in a short time (less than 1 h). In fact, the same research group described later the rapid and simultaneous detection of four different food-contaminating pathogenic bacteria (*Salmonella* spp., *Lysteria monocytogenes*, *Escherichia coli* O157:H7, and *Staphylococcus aureus*) using this methodology [12]. The analysis of non-specific amplicons and mixtures of different complementary sequences, after modification of each addressable electrode in the array with a different capture probe, confirmed the selectivity of the procedure with negligible cross-hybridization or interference. Although this device is not validated with real samples, it can be considered as a further step towards the design of easy to use tools for screening analysis of nucleic acids. The authors expect these arrays to further improve the reliability, speed and cost-effectiveness of the hybridisation-based detection of nucleic acids.

The implementation of sample preparation, DNA amplification, and electrochemical detection in one silicon and glass-based microchamber, and its application for the multiplexed detection of *Escherichia coli* and *Bacillus subtilis* cells was reported by Yeung *et al*. [22]. The microdevice incorporated a thin-film heater and temperature sensor patterned on the silicon substrate. An ITO electrode array was constructed within the microchamber as the transduction element. Oligonucleotide probes specific to the target amplicons were individually positioned on each ITO surface by electrochemical copolymerization of pyrrole and pyrrole-probe conjugate. The identification of the two model pathogens involved the following steps: (1) sample preparation by thermal cell lyses and magnetic particle-based target genome isolation; (2) target DNA amplification by PCR; (3) hybridization of the amplicons to their complementary oligonucleotide capture probes immobilized onto individual electrode surfaces, and (4) electrochemical transduction of the recognition event via gold nanoparticles with signal amplification using electrocatalytic silver deposition. The portable electrochemical instrumentation as well as the simple microchip design were considered to allow on-site pathogen detection. However, only cell culture samples were tested.

A general limitation for multiplexed electrochemical detection is the technical difficulty to obtain an output from a multielectrode array due to wiring complexity and requirement for a multi-channel reader. Therefore, most of modern microarray platforms are based on fluorescent detection. CombiMatrix core technology is a platform that permits in obtaining an electrical signal output by semiconductor addressing, and thus, to use both fluorescent and electrochemical approaches. Ghindilis *et al*. [23] have developed and checked the performance and limitations of the CombiMatrix oligonucleotide microarray platform that contains 12,544 individually addressable microelectrodes in a semiconductor matrix (ElectraSense™ 12K microarray platform). The approach is based on the detection of redox active chemistries proximal to specific microarray electrodes. Each working electrode is made of high purity Pt with a circular geometry (44 μm diameter), and coated with a bio-membrane, or porous reaction layer. The working electrodes are surrounded with a Pt grid used as counter electrode and the voltage between the working and the counter electrode is set at 0 V. The ElectraSense™ Reader is able to detect current values for the whole array in approximately 25 s, with each electrode being read for less than 2 ms. Oligonucleotide probes were immobilized on the electrodes for the specific capture of biotin labeled target molecules from the hybridization solution. This bound target was subsequently labeled with HRP using biotin-avidin chemistry. HRP catalyzed the oxidation of tetramethylbenzidine (TMB) and, due to the close proximity of HRP to the electrode surface, the oxidized TMB was readily reduced by application of a reducing potential. The ElectraSense™ platform was compared to the standard fluorescent detection, and good consistency was obtained between the two detection modes. The electrochemical approach showed lower detection limits (0.75 pM as compared to 1.5 pM with fluorescent detection), lower cost, and higher operational convenience, thus demonstrating to be valid as a promising alternative to the fluorescent detection.

The ElectraSense™ platform was used to develop nucleic acid assays for genotyping of multiple pathogens including bio-threat agents (such as *Bacillus anthracis*, *Yersinia pestis*, and other microorganisms including *Escherichia coli* and *Bacillus subtilis*) and common pathogens of the respiratory tract, such as influenza A virus.

The same microarray platform and detection scheme were also used for genotyping identification of upper respiratory tract pathogens [24]. In this case, the assay was developed to detect four bacterial pathogens (*Bordetella pertussis*, *Streptococcus pyogenes*, *Chlamydia pneumoniae*, and *Mycoplasma pneumoniae*) and nine viral pathogens. The use of multiple probes in a flexible platform allowed testing probes empirically and then selecting highly reactive probes for further iterative evaluation. Also, as a consequence of the enzyme catalyzed electrochemical detection that can be read directly from the array, there was no need for image analysis or for expensive and delicate optical scanning equipment. The combination of assay speed (approximately 1 h hybridization), array sectoring (which would allow multiple assays on one array), the potential to strip and reuse the chip up to five times, and the adaptability to inexpensive electrochemical scanning devices make these arrays a superb adjunct to real-time PCR by supporting multiplex assays and analyses in a single PCR tube. Nevertheless, the methodology has not been applied to the analysis or real food samples.

Goto *et al*. [25] developed a microfabricated electrochemical DNA chip for the specific and quantitative detection of PCR products from bacterial and viral infected mice samples. The chip did not require DNA labeling, and the hybridization signal could be detected as an anodic current. The chip consisted of 40 working electrodes (200 μm diameter), a reference and a counter electrode. Oligonucleotide probes with a thiol group at the 5′-ends were spotted on the gold working electrodes surface and the chip was kept at room temperature for 1 h to immobilize the probes by chemisorption. Hybridization of the PCR products was carried out at 35 °C for 5 min and, after a washing step, the chip was reacted with 50 μL of 20 mmol L^−1^ phosphate buffer (pH 7.0) containing 5 μmol L^−1^ Hoechst 33258 and 100 mmol L^−1^ NaCl for 30 s at 25 °C. The electrochemical signals for each electrode were measured by cyclic voltammetry (−100 – 900 mV) in the phosphate buffer. The system could detect 10^−2^ cfu of *Clostridium piliforme*, 1 cfu of *Helicobacter bilis*, 10^−1^ cfu of *Helicobacter hepaticus*, and 1.6 cfu of mouse hepatitis virus. It should be noted that, in this case, the novel electrochemical DNA chip developed was successfully applied to the specific and quantitative detection of PCR products from bacterial and viral infected mice samples.

The electrochemical detection of microbial contaminants using commercially available, hand-held instruments was reported by LaGier *et al*. [26]. Multi-target capability was demonstrated with an 8-plex assay for bacterial and viral targets using isolated DNA, natural beach water spiked with human feces, and water and sediments collected from New Orleans (Louisiana, USA) following Hurricane Katrina. The multi-target assay was used for the determination of *Enterococcus* spp, used to indicate the presence of fecal pollution in environmental waters; the human-specific HF8 cluster of *Bacteroides*; the *esp* gene from *Enterococcus faecium*, which is used as a proxy for human fecal pollution; the water-borne bacterial pathogens *Escherichia coli* O157:H7, *Salmonella* spp., *Campylobacter jejuni*, and *Staphylococcus aureus*; and adenoviruses known to cause disease in humans via a waterborne route of transmission. For the sample analysis, amplicons were labeled with biotin on one end and fluorescein on the other during PCR. Eight-well carbon sensor strips were used where each surface was exposed to a different microbial target. Thus, following PCR, 1 μL of each amplicon was diluted in 100 μL of nuclease-free water. Then, 40 μL of diluted amplicons were placed on the pre-washed sensors and allowed to bind for 5 min at room temperature. After washing, the sensor was incubated for 5 min with 50 μL of HRP-conjugated antifluorescein antibody (0.75 U μL^−1^) at room temperature. The electrochemical reaction was initiated by adding 50 μL of HRP substrate and intermittent pulse amperometry (IPA) readings were obtained in approximately 30 s. The method could be used to rapidly (3–5 h) screen environmental water samples for the presence of microbial contaminants and have the potential to be integrated into semi-automated detection platforms. To our knowledge, this is the first time that multiplexed detection of pathogen microorganisms is applied to real environmental samples including method validation by testing laboratory samples as well as beach water spiked with human feces. However, the PCR and electrochemical steps of the analysis need to be integrated into a single device to be more practical for field applications.

Pöhlmann *et al*. [27] used esterase 2 (EST2) from *Alicyclobacillus acidocaldarius*, a thermostable reporter enzyme for the detection of foodborne bacteria by one-step rRNA/DNA hybridization between a bacterium-specific capture oligodeoxynucleotide (ODN), bacterial 16S rRNA and a uniform EST2-ODN reporter conjugate. The electrochemical biochip used (11 mm × 13 mm) consisted of eight individual electrodes, four of them used as reference. A 10 μL U-shaped flow chamber was placed over the electrodes and the printed circuit board of the chip was connected to a multipotentiostat device attached to a PC. The identification of 16S rRNA by electrochemical detection was based on the simultaneous hybridization between the capture ODN immobilized on a gold electrode via thiol-gold-linkage, helper ODN and detector EST2-ODN conjugate with the corresponding regions of the 16S rRNA. In this way, the reporter EST2 was immobilized in the vicinity of the electrode. The subsequent enzymatic hydrolysis of p-aminophenylbutyrate resulted in the production of p-aminophenol (pAP) as electroactive substrate leading to a detectable electrical signal. In this system, the hybridization event was amplified through the high turnover of the enzymatic reaction and also through redox recycling of quinonimine to pAP. A detection limit of 500 cfu *Escherichia coli* was reported together with the possibility to discriminate two Gram-negative and two Gram-positive bacteria, which demonstrated the method specificity and its potential for parallel detection of microorganisms. The method was applied to the determination of *E. coli* in an endogenously infected meat juice sample; the results obtained demonstrated the possibility for detection of microorganisms in contaminated food samples within one working day.

Although a number of multiplexed electrochemical systems have been described for the determination of pathogen microorganisms based on DNA samples, to date only a few of them have been applied to the analysis of real samples. A lot of work is still to be done in order to integrate these methodologies in single devices able to accomplish the detection of multiple bacteria in field samples.

## 4. Other Electrochemical Approaches

The simultaneous identification of various pathogen microorganisms has been also addressed by means of other electrochemical approaches different that measuring antibody-antigen or hybridization reactions.

Ertl *et al*. [28] described an electrochemical biosensor array in which the transduction process was based on respiratory cycle activity measurements, where the microorganism’s native respiratory chain is interrupted with non-native external oxidants, only viable cells being detected this way. Lectins were employed as selective recognition elements to distinguish six microbial species (*Baccilus cereus*, *Staphylococcus aureus*, *Proteus vulgaris*, *Escherichia coli*, *Enterobacter aerogenes*, and *Saccharomyces cerevisiae*). Lectins were immobilized onto various membrane surfaces by adsorption with and without intermolecular cross-linking, through avidin-biotin anchors using biotinylated lectins and by covalent coupling to activated membrane surfaces. Lectin-modified membranes were incubated in pure suspensions of microorganisms to allow selective cell attachment and fixed on the surface of a Pt electrode. Chronocoulometric measurements were used for the assessment of lectin-cell binding. They were made in a buffer containing succinate, formate, and ferricyanide and menadione as the redox mediators, for an array of ten lectin-modified membranes, with each of the six microorganisms. Factor-based principal component analysis (PCA) was used to analyze the chronocoulometric data. The obtained results showed groupings of replicate measurements for the six microorganisms, suggesting that rapid bacteria identification was possible using an array of lectin-modified electrodes. Later on, these authors used the same transduction principle, although using an electrochemical screen-printed biosensor array, for the recognition of four *E. coli* subspecies after 40 min total analysis time [29]. Ten different lectins were separately immobilized onto porous surface-activated membranes that were then exposed to untreated *E. coli* cultures for 30 min, rinsed, and layered over the individual screen-printed carbon electrodes of the sensor array. The reagent solution containing the oxidants menadione and ferricyanide, as well as the respiratory substrates succinate and formate was added to each well in the sensor array and incubation was let to proceed at 37 °C for 5 min. Electrochemical oxidation of ferrocyanide for 2 min provided chronocoulometric data related to the quantities of bound cells. These screen-printed sensor arrays were used in conjunction with factor analysis for the rapid identification of *E. coli* B, *E. coli* Neotype, *E. coli* JM105 and *E. coli* HB101. The systematic examination of the lectin binding patterns showed that these four *E. coli* subspecies are readily distinguished using only five essential lectins. Since lectins are readily available and inexpensive, this type of biosensor array showed great promise for microbiological identification in real samples. However, the studies carried out in these papers were only applied to cell cultures. The application of this methodology to complex matrices such as food products would need the coupling of a sample treatment procedure in order to remove compounds that could interfere with lectin-cell binding.

A traditional automated bacterial detection method is the one based on the changes in the electrical characteristics of a medium where bacteria are cultured. These changes are produced by the release of ionic metabolites from live metabolizing cells, monitored over time through the ac impedance of a pair of electrodes immersed in the culture medium. If the impedance changes beyond a certain threshold, a positive detection is indicated. However, the detection time of the conventional impedance-based method can be quite long when the number of bacterial cells present in the sample is very small. The lower the initial concentration of microorganisms, the longer it takes for impedance to change by a measurable amount. Rapid detection of a few live cells (1–10) is, however, possible if the cells are confined into a volume of the order of nanoliters. Gómez *et al*. [30] described a microscale impedance-based technique for detecting the metabolic activity of a few live bacterial cells. The method used a microfluidic prototype consisting of a network of channels and chamber etched in a crystalline silicon substrate. The complex impedance of bacterial suspensions was measured at interdigitated Pt electrodes in a 5.27 nL chamber at frequencies between 100 Hz and 1 MHz. After 2 h of off-chip incubation, the minimum number of live cells suspended in a low conductivity buffer that could be distinguished from the same number of heat-killed cells was of about 100 *Listeria innocua*, 200 *L. monocytogenes*, and 40 *Escherichia coli* cells. Although the authors mention the possibility to apply the developed methodology to the analysis of real samples, to our knowledge this step has not been undertaken till today. In this case, a microscale detection system where the target bacteria could be selectively captured and incubated inside the biochip would be necessary. The fact that heat-killed cells generate small changes in impedance resulting in a large number of dead cells producing the same as or larger signal than a small number of live cells should also be taken into account.

**Figure 3.**
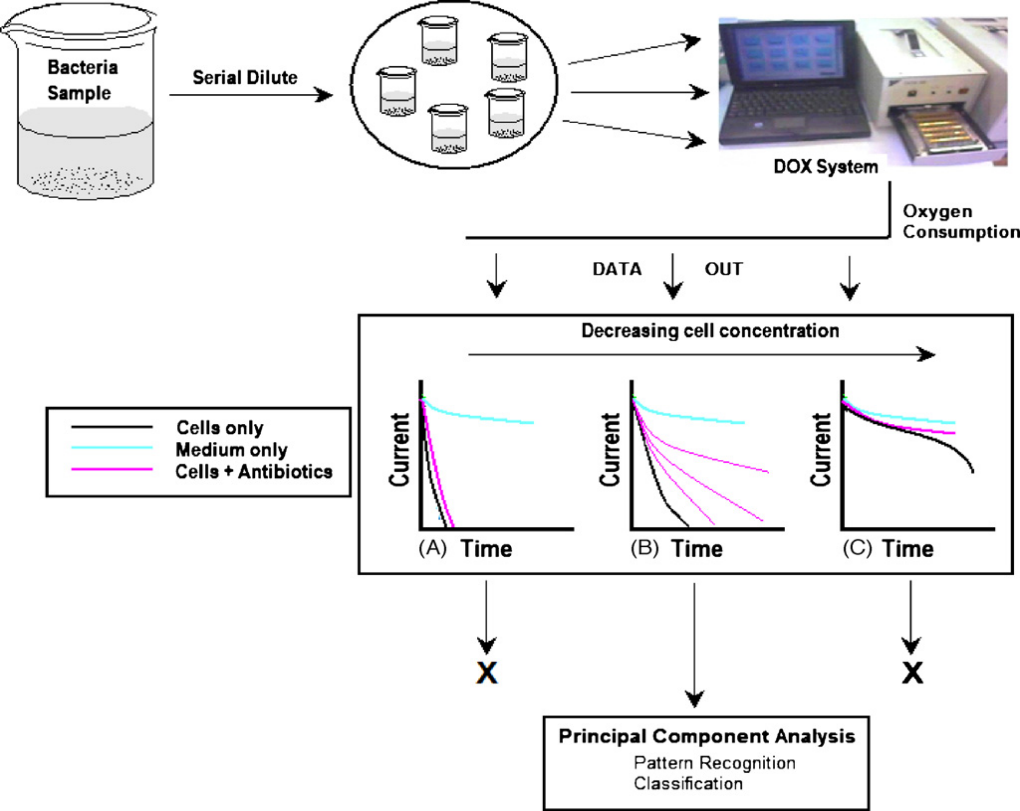
DOX-PCA concept [32]. (A–C) represent DOX responses for high, medium and low cell concentrations, respectively.

The integration of a fully autonomous electrochemical biosensor with pattern recognition techniques for the detection and classification of bacteria at subspecies and strain levels was described by Karasinski *et al*. [31]. The classification scheme described in this work was based on the hypothesis that, under identical experimental conditions, various bacteria consume oxygen at different rates and are affected in different ways by selected antibiotics. A system consisting of a 96-well-type electrodes array (DOX-dissolved oxygen sensor) coupled with principal components analysis (PCA) was used to analyze and classify *Corynebacterium glutamicum*, *Microcuccus luteus*, *Staphylococcus epidermis*, *Yersinia ruckeri*, *Escherichia adecarboxylata*, *Comamonas acidovorans*, *Bacillus globigii*, and three strains of *Escherichia coli* (K12, SM10, ATCC 25922). Two experimental strategies were employed with the DOX-PCA system. The first one consisted of monitoring the rate of bacteria respiration, quantified by the DOX electrochemical sensor as a measure of oxygen consumed by the cells over time, while the second strategy was based on monitoring changes in cell respiration as a result of the effects induced by the presence of different antibiotics (tetracycline, chloramphenicol and ampicillin). Antibiotics may be lethal to the cells, completely stopping cell respiration or division, they can reduce the rate of cell division, or they can have no effect at all. These effects were studied by comparing the oxygen consumption curves of bacteria with and without an antibiotic. The large amounts of data generated with the DOX multisensor were treated by PCA which allowed searching and identifying a microbial species in a database of well-characterized bacteria. Since the method did not require culturing bacteria, additional reagent, or incubation time, it could be used for screening. Moreover, the same test could be used for obtaining practical information on the type, resistance, and dose of antibiotic necessary to establish optimum diagnosis, treatment, and decontamination strategies. More recently, the same research group used the amperometric signals generated by the 96-well multi-array DOX-PCA for continuous monitoring, identification and differentiation of five bacteria: *Escherichia coli*, *Escherichia adecarboxylata*, *Comamonas acidovorans*, *Corynebacterium glutamicum* and *Staphylococcus epidermidis*, including various concentrations of cells [32] (see Figure 3).

A new application for microbial biosensors, the rapid diagnosis of soil-borne diseases, has been reported recently by Hashimoto *et al*. [33]. The developed system consisted of two biosensors constructed using equal quantities of two different microbes immobilized onto a nitrocellulose filter which was attached to the surface of an oxygen electrode. The two sensors were coupled as a dual sensor system and used for simultaneous measurements carried out by immersing them in a soil extract. When microbial respiration increased due to the assimilation of organic compounds in the sample, the decrease of the dissolved oxygen concentration was measured. The ratio of responses to non-diseased soils was higher than that to infested soils. The biosensor system was used to investigate the effect of six antagonists on the inhibition of four diseases. The ratio between the responses of the two sensors correlated with symptoms, except for two samples where the antagonist promoted the disease’s development. Given that the ratios of both sensors’ responses were related with the antagonists ability to protect against diseases development, further improvements in the described methodology could lead to a future where the sensors’ responses can be used as indicators for the screening of biological control agents.

## 5. Global Overview on the Existing Electrochemical Methodologies for Multiplexed Detection of Foodborne Pathogens

Table 1 summarizes the characteristics of the different methodologies reported in literature for the electroanalytical multiplexed detection of foodborne pathogen microorganisms. As can be seen, although there are already several systems described for this purpose, only a few of them were actually validated or applied to the determination of these microorganisms in real samples. On the contrary most of them were only applied in pure cultures of the considered organisms. As a consequence, currently there is still a lot of work to be done in order to fulfill nowadays requirements for this kind of analysis in order not to have their use limited to research laboratories.

It is well-known that the main drawback of traditional pathogen detection methods is that, although they are sensitive enough, they are too slow. Techniques using fluorescent detection, surface plasmon resonance (SPR) or quartz crystal microbalance (QCM) have been widely studied but, although showing a good sensitivity, they also have several disadvantages such as high cost of the instrumentation, low speed, and the difficulty with portability, miniaturization or on-site analysis which makes them unattractive to end users. Although optical techniques can nowadays provide better sensitivity than electrochemical ones, the cost of electrochemical techniques is much lower, they are much easier to use, able to operate in turbid media, and amenable to miniaturization and to the creation of new compact, portable and hand-held designs for field use. Accordingly, further development of sensing platforms based on those previously described in literature but including the application to real samples is of great interest for on-site monitoring applications. As soon as the possibility to apply these methodologies to real samples is demonstrated and validated, on-site electroanalytical detection and measurement in the field will become more widely used for applications in health monitoring.

## 6. Conclusions

The implementation of multianalyte methodologies implies significant advantages over single analyte tests in terms of cost per assay, work loading, assay throughput and suitability, and means a major trend in current Analytical Chemistry. As a general strategy, electrochemical approaches for the multiple and simultaneous detection of pathogenic bacteria involve bioplatforms and devices making use of principles and methodologies from immuno- and genosensors, as well as from other approaches such as the monitoring of oxygen consumption coupled to computerized data analysis.

Immunoelectrochemical multiplexed platforms, although still with few applications for the simultaneous detection of pathogenic bacteria, demonstrate a versatility which promises the near solution of relevant multi-analyte problems. On the other hand, the low cost, pathogen specificity similar to the plate culture and high operational convenience of multiplexed detection based on DNA platforms, make them as promising alternatives to the common fluorescent detection in this field. Moreover, the electrochemical immuno- and DNA platforms have demonstrated to provide the specificity to distinguish the target pathogen in a multiorganisms matrix, the adaptability to detect different analytes, the sensitivity to detect bacteria on-line without pre-enrichment or pre-concentration steps, and the rapidity to give real-time results.

The development of automated electrochemical microarray platforms constitute nowadays a great challenge which should focus even more on engineering aspects such as the optimization of user interfaces and sample handling, the use of micro- and nano-fabrication techniques enabling the performance of multi-analyte analysis with the same device, the development of parallel computational methods to convert electronic responses for each analyte into concentration data, and the integration of these bioplatforms into portable systems.

## Figures and Tables

**Table 1.** Electrochemical multiplexed detection of foodborne pathogens.

**Methodology**	**Analytes**	**Sample**	**Analysis time**	**Detection limit**	**Ref.**
Impedance-based fieldable immunosensor	*E. coli* O157:H7 *Salmonella* spp	-	Response time < 1 min	10 cfu *E. coli* O157:H7	[15]
Impedance-based HRP-labelled immunosensor	Rat IgG, HBsAg, HBeAg	-	-	10 pg mL^−1^ HBsAg	[16]
Ag-PSA-based DNA sensors	*Stachybotrys chartarum* *Escherichia coli*	-	-	2.0 × 10^−12^ M oligonucleotides	[18]
IDA-AP amplification-based RNA microarray sensors	*Escherichia coli*, *Pseudomonas aeruginosa*, *Enterococcus faecalis*, *Staphylococcus aureus*, *Staphylococcus epidermidis*	-	Fully automated detection in less than 25 min	0.5 ng μL^−1^ (16 fmol) *E. coli* RNA	[13]
IDA-β-Gal amplification-based DNA microarray sensors	*Bacillus cereus*	-	-	-	[19]
IDA-AP amplification-based DNA microarray sensors	*Bacillus anthracis*, *Yersinia pestis*, *Francisella tularensis* and ortho pox viruses	-	Fully automated detection in less than 27 min	-	[20]
SPE-AP amplification-based array DNA sensors	*Listeria monocytogenes* toxin inlA	-	Total analysis time < 1 h	0.75 nM	[21]
SPE-AP amplification-based array DNA sensors	*Salmonella* spp., *Lysteria monocytogenes*, *Escherichia coli* O157:H7, *Staphylococcus aureus*	-	Total analysis time < 1 h	-	[12]
Ag-PSA-based DNA sensors	*Escherichia coli* *Bacillus subtilis*	-	-	-	[22]
HRP-amplification-based DNA microarray sensor	*Bacillus anthracis*, *Yersinia pestis*, *Escherichia coli* and *Bacillus subtilis*	-		0.75 pM	[23]
HRP-amplification-based DNA microarray sensor	*Bordetella pertussis*, *Streptococcus pyogenes*, *Chlamydia pneumoniae*, and *Mycoplasma pneumoniae*	-	-	2 fg *M. pneumoniae*	[24]
Hoechst 33258-based DNA array sensor	*Clostridium piliforme*, *Helicobacter bilis*, *Helicobacter hepaticus*, and mouse hepatitis virus	Mice caecum, faeces, heart and liver	-	10^−2^ cfu *C. piliforme*	[25]
HRP-amplification-based DNA multiwell sensor strips	*Escherichia coli* O157:H7, *Salmonella* spp., *Campylobacter jejuni*, and *Staphylococcus aureus*	Natural beach water spiked with human faeces, and water and sediments collected from New Orleans (LA, USA) following Hurricane Katrina	3 – 5 h	≤ 1,000 cells *Karenia brevis*	[26]
Esterase 2-amplification-based DNA array sensor	*Escherichia coli, Bacillus subtilis, Bacillus atrophaeus, and Listeria innocua*	Meat juice	One working day	500 cfu *E. coli*	[27]
Chronocoulimetric respiratory cycle activity measurements and PCA chemometric data treatment-based methodology	*Baccilus cereus*, *Staphylococcus aureus*, *Proteus vulgaris*, *Escherichia coli*, *Enterobacter aerogenes*, and *Saccharomyces cerevisiae*	-	-	-	[28]
Chronocoulimetric respiratory cycle activity measurements and PCA chemometric data treatment-based methodology	*Escherichia coli* B, *Escherichia coli* Neotype, *Escherichia coli* JM105 and *E.coli* HB101	-	Total analysis time 40 min	-	[29]
Microscale impedance-based metabolic activity detection-based methodology	*Listeria innocua*, *L. monocytogenes*, and *Escherichia coli*	-	2 h	100 *L. innocua*, 200 *L. monocytogenes*, and 40 *E. coli* cells	[30]
Electrochemical oxygen multisensor array and PCA chemometric data treatment-based methodology	*Corynebacterium glutamicum*, *Microcuccus luteus*, *Staphylococcus epidermis*, *Yersinia ruckeri*, *Escherichia adecarboxylata*, *Comamonas acidovorans*, *Bacillus globigii*, and three strains of *Escherichia coli*	-	-	-	[31]
Electrochemical oxygen multisensor array and PCA chemometric data treatment-based methodology	*Escherichia coli*, *Escherichia adecarboxylata*, *Comamonas acidovorans*, *Corynebacterium glutamicum* and *Staphylococcus epidermidis*	-	8 h	1×10^6^ cfu mL^−1^	[32]
Electrochemical oxygen dual sensor	*Ralstonia solanacearum, F. oxysporum* f. sp. *Lactucae* strain SN3B*, Plasmodiophora brassicae,* and *F. Oxysporum* f. sp. *spinaciae*	Soil	-	-	[33]
